# Multifaceted Analysis of *Lactobacillus plantarum* DMR14 Reveals Promising Antidiabetic Properties Through In Vivo Assays and Molecular Simulations

**DOI:** 10.1111/jcmm.70347

**Published:** 2025-01-26

**Authors:** Shirmin Islam, Suvro Biswas, Md. Ariful Islam, Jui Biswas, Amit Kumar Dutta, Golam Gaus Mohiuddin, Md. Abu Saleh, Shahriar Zaman

**Affiliations:** ^1^ Microbiology Laboratory, Department of Genetic Engineering and Biotechnology University of Rajshahi Rajshahi Bangladesh; ^2^ Department of Microbiology University of Rajshahi Rajshahi Bangladesh; ^3^ Department of Pharmacy Noakhali Science & Technology University Sonapur Noakhali Bangladesh

**Keywords:** diabetes, GC–MS, *Lactobacillus plantarum*, molecular docking, molecular dynamics simulation

## Abstract

Due to the growing concern about diabetes worldwide, we investigated the antidiabetic potential of *Lactobacillus plantarum* DMR14, assessing its effects on the diabetic mice and identifying safe, bioactive compounds targeting DPP4 protein for drug development through various methods, including in vivo assays, GC–MS analysis and molecular docking simulations. The animal experiments showed that after 3 weeks of treatment, the blood sugar levels of mice given the bacteria were reduced by 35.03% compared to baseline. The treatment also significantly lowered blood lipids such as triglycerides, total cholesterol and LDL cholesterol, but did not affect HDL cholesterol levels. Additionally, we identified three compounds that effectively targeted a protein (DPP4) involved in diabetes (PDB ID: 4A5S). These compounds were predicted to be safe for absorption, processing and elimination by the body, and showed no signs of inducing cancer in computer simulations. Further simulations indicated that these compounds bind stably to the protein over time. Diabetic mice treated with *Lactobacillus plantarum* DMR14 exhibited improved organ health, reduced glucose levels and better metabolic markers. Computer analysis suggested compounds that could enhance enzyme inhibition, indicating potential antidiabetic properties in this strain. These suggested compounds could be considered potential candidates for developing antidiabetic drugs.

**Abbreviations**
3D: three dimensional
ADMET: absorption, distribution, metabolism, excretion and toxicity
AMBER14: assisted model building with energy refinement 14
CFU: colony‐forming unit
CID: compound identifier
DNS: 3,5‐dinitrosalicylic acid
DPP‐4: dipeptidyl peptidase‐4
fs: femtoseconds
GC–MS: gas chromatography–mass spectrometry
GI: gastrointestinal
HDL: high‐density lipoprotein
HDL‐C: high‐density lipoprotein cholesterol
LDL: low‐density lipoprotein
LDL‐C: low‐density lipoprotein cholesterol
Lp: lipoprotein
mmff94: merck molecular force field 94
MRS broth: de Man, Rogosa and Sharpe broth
NF‐kB: nuclear factor‐kappa B
OD: optical density
PDB: protein data bank
PDBQT: protein data bank with partial charge (Q) and atom type (T)
PME: particle mesh Ewald
RDMS: root mean square deviation
Rg: radius of gyration
RMSF: root mean square fluctuation
SASA: solvent‐accessible surface area
SDF: spatial data file
SPSS: statistical package for the social sciences
STZ: streptozotocin
T2D: type 2 diabetes
T2DM: type 2 diabetes mellitus
TC: total cholesterol
TG: triglycerides
TIP3P: transferable intermolecular potential with 3 points
TPSA: topological polar surface area
YASARA: yet another scientific artificial reality application

## 1 Introduction

Diabetes is a condition that affects how your body processes sugar (glucose) in the blood. It is caused by either the body not using insulin properly or not producing enough insulin. High blood sugar levels left untreated can lead to long‐term health problems [1]. Around 90%–95% of people with diabetes have type 2 [2]. Cases of type 2 diabetes are rising rapidly, with projections suggesting there could be over 629 million people affected by 2045 [3]. Because of its impact on blood sugar control, diabetes is linked to a variety of serious health problems. These complications can include blindness, stroke, heart attack, kidney failure, even pancreatic cancer and amputation of the lower limbs [4, 5]. So, the ultimate objective is to achieve primary prevention and efficient treatment of the disease for the general people.

Though some drugs are available to treat diabetes, its harmful side effects like diarrhoea, fatigue, drug resistance, and kidney and liver are making a thirst for developing new drugs against it. Furthermore, for getting convenient solutions from sources, researchers are going back to nature to meet up the problem [6]. The human gut microbiome serves various physiological functions such as nutrient extraction, vitamin synthesis, immune modulation, digestion, metabolism and protection against pathogens [7]. Recent research suggests a connection between the health of gut bacteria and certain conditions like obesity and type 2 diabetes. Mechanisms linked to gut barrier dysfunction can trigger inflammation, which contributes to metabolic disorders. The trillions of bacteria living in our gut, called gut microbiota, seem to influence how easily substances pass through our intestines and how our body fights off invaders. These factors are important in the development of type 2 diabetes [7, 8, 9]. Studies have shown that probiotics can help lower blood sugar levels [10]. This is why researchers are exploring the use of probiotics and fermented foods as new approaches to managing type 2 diabetes [11]. Several studies have found that certain lactic acid bacteria, including *Lactobacillus plantarum* , *Lactobacillus reuteri* , *Lactobacillus rhamnosus* and *Lactobacillus brevis* strains, may be helpful in managing diabetes [12, 13]. According to recent studies, *Lactobacillus plantarum* has been utilised as a probiotic to alleviate symptoms of diarrhoea, bowel syndrome and metabolic syndrome, with a particular focus on hyperglycaemia. These studies propose that using *Lactobacillus plantarum* as a supplement could help prevent diabetes by regulating blood glucose levels and improving insulin sensitivity [14, 15].

Researchers led by Yin Liu investigated the gut bacteria in people who consumed fermented dairy products. They studied how these bacteria might influence certain cellular pathways and looked for specific bacteria that could potentially benefit blood sugar management [16]. Further research, including animal studies and cell experiments, has shown *Lactobacillus plantarum* YJ7 to be a promising candidate as a substance that can lower blood sugar. Researchers recommend exploring its use in food and pharmaceutical products to potentially help prevent diabetes [17].

Drug discovery is becoming possible with the development of computational studies. In silico research is now being conducted to find new compounds for a more potent antidiabetic agent to find a more promising treatment against this massive disease [18, 19]. However, no work was carried out to identify potential compounds from bacterial strains through in vivo studies. So, the goal of our present work was to identify potential antidiabetic compounds from *Lactobacillus plantarum* DMR14 through in vivo and in silico studies.

## 2 Methods and Materials

### 2.1 Isolation of *Lactobacillus plantarum* DMR14 From Fermented Oats

Quaker oats samples were collected from a supermarket in Shaheb‐bazar, Rajshahi, Bangladesh, and fermented in MRS broth for 24 h at 37°C to isolate *Lactobacillus plantarum* DMR14. After fermentation, the sample was serially diluted, and 100 μL from each dilution was plated onto MRS agar to isolate individual colonies, which were incubated aerobically at 37°C for 24–48 h. Distinct colonies were selected, subcultured for purity and grown in MRS broth at 37°C. Glycerol stocks were prepared for long‐term storage at −80°C. Molecular identification of the isolate was carried out by extracting genomic DNA, amplifying the 16S rRNA gene through PCR and confirming the strain as *Lactobacillus plantarum* using sequencing and BLAST analysis [20].

### 2.2 In Vivo Anti‐Diabetes Activity

Animals and experimental procedures were conducted on Swiss albino mice (male) *Mus musculus* (28–30 g). They were kept in a controlled environment with consistent temperature and light/dark cycles for a week before and during the experiment. The mice had constant access to food and water. To induce type 2 diabetes, we fed the mice a high‐fat diet for 3 weeks, followed by four daily injections of a low‐dose medication (streptozotocin: 40 mg/kg i.p.) in the fourth week. The high‐fat diet used in this study was specifically formulated to induce obesity in the animal model, providing 60% of its caloric content from fat, with the remaining 40% derived from carbohydrates and protein. The fat source was primarily beef tallow, while casein was used as the protein source and maltodextrin as the carbohydrate source. The diet also included essential micronutrients such as vitamins (A, D3, E, K, C and B complex), minerals (calcium carbonate, sodium chloride, potassium chloride and magnesium sulphate) and other nutrients like cellulose for fibre content. The specific composition of the diet was obtained from Xietong Bio‐engineering Co. Ltd. China (HFD, XTHF60).

After 3 days, we checked the mice's blood sugar levels [21]. Only mice with high blood sugar were included in the experiment and divided into four groups (Table 1). We monitored the mice's food intake, weight and blood sugar levels throughout the experiment, recording data on specific days (Table 1). Before the oral administration of a bacterial suspension, specifically *L. plantarum* DMR14 isolated from fermented oats, mice underwent a fasting period lasting between 2 and 4 h. This bacterium was selected for the experiment because it is a probiotic species that aligns with the objectives of our research and is part of the sequenced bacterial strains available in our laboratory. The suspension was meticulously prepared to achieve an optical density (OD) approximating 1, containing approximately 3.5×10^9^ cfu/mL. This dose was selected based on existing literature, practical considerations and the results of our dilution study. Mice were administered various concentrations of *L. plantarum* DMR14, with CFU doses ranging from 1.5×10^8^ to 5.5×10^9^ CFU/mL, which aligns with common dosages used in probiotic studies targeting glucose metabolism. The 3.5×10^9^ CFU/mL dose was found to be most effective in reducing blood glucose levels, with no adverse effects on health or behaviour. Higher doses (5.5×10^9^ CFU/mL) did not show additional therapeutic benefits, confirming the optimal dose. This dilution approach helped identify the most effective concentration for *L. plantarum* DMR14. Moreover, in probiotic research, bacterial concentrations around 10 [9] to 10 [10] CFU/mL are commonly used to ensure that the viable bacteria administered can produce a measurable effect on the host's physiology, such as gut microbiota modulation and immune response [22]. The dosing volume was standardised at 10 mL/kg, with the precise amount administered varying between 300 and 350 μL, contingent upon the size of the respective mouse. The suspension and metformin (positive control) were introduced directly into the stomach via an oral gavage needle, ensuring aseptic conditions were maintained throughout the procedure. We tracked how much the mice ate and weighed them regularly. We also checked their blood sugar levels on specific days throughout the experiment, including the 1st, 7th, 14th and 21st days of treatment (Table 1).

**TABLE 1:** Grouping of experimental mice.

Groups	Groups treated as	No. of mice	Dose mg/kg body weight	Dose mg/kg body weight
Group I	Normal control	6	They received citrate buffer (pH‐ 4.5) intraperitoneal	Normal diet
Group II	Diabetic control	6	Mice were administered STZ	Normal diet
Group III	Standard drug	6	Mice were administered STZ	Normal diet + Metformin (orally 100 mg/kg body weight)
Group IV	Experimental Bacteria	6	Mice were administered STZ	Normal diet + Bacteria (orally 3.5×10^9^ cfu/mL single dose was used as treatment).

### 2.3 Biochemical Analysis

Following the completion of the 21‐day treatment protocol, the mice underwent an extended period of fasting overnight, after which they were humanely euthanised via the inhalation of anaesthetic (isoflurane). The blood samples were taken from the mice's hearts and stored them in special tubes at a cold temperature (4°C). After the blood clotted, they spun the samples in a centrifuge to separate the clear liquid part (plasma) at another cold temperature (4°C) for 15 min. Then, they carefully removed a different liquid part (serum) from the plasma and stored it in separate tubes at a very cold temperature (−80°C) for further testing. The serum lipid profile is pivotal in evaluating cardiovascular disease risks and the metabolic status of diabetic patients. High levels of total cholesterol, triglycerides and LDL cholesterol are linked to a greater risk of heart disease. In contrast, HDL cholesterol helps protect against heart disease by removing cholesterol from the arteries. Assessing these components typically employs enzymatic techniques using kits. For example, the cholesterol oxidase method measures TC by oxidising free cholesterol, producing a detectable colour or fluorescence. Similarly, triglycerides are assessed through the glycerol phosphate oxidase method, which also results in a colour change upon enzymatic action. LDL and HDL concentrations are determined using precipitation followed by enzymatic quantification or direct assays [23, 24]. Moreover, organs like liver, kidney, pancreas and heart were collected carefully and weighed through an electric balance. Lastly, histopathological tests were conducted with the organs for getting the relationship with the biochemical test results.

### 2.4 Histopathological Preparation

For histopathological analysis, the collected organs were preserved in formalin to maintain tissue integrity. Tissue collection occurred at the end of the 21‐day treatment protocol, followed by an overnight fasting period. After euthanising the mice, organ harvesting was performed, and the tissues underwent standard histopathological processing, which included dehydration, clearing and embedding in paraffin wax to prepare them for sectioning. Tissue sections were cut at a thickness of 5–10 μm and stained with Haematoxylin and Eosin (H&E) or other appropriate stains to highlight structural changes. The stained sections were then examined under a light microscope to identify histopathological alterations.

### 2.5 GC–MS Sample Preparation

The optimal SPME experimental settings were developed based on prior investigations of Chen et al. [25]. In a 15‐mL vial closely closed with a PTFE–silicon septum, about 40 mL of bacterial culture and 1.5 g of NaCl were mixed at 80 rpm in a 15‐mL vial tightly capped with a PTFE–silicon septum and the taste components in bacterial strains are generated during the extractives process and during equilibrium. As a result, the extractives’ temperature was set at 40°C. The pretreatment (conditioned at 27°C for 30 min) SPME fibre was put into the headspace after the vial holding the sample was equilibrated at 40°C for 10 min on a heating platform agitation, and extractives were done for 30 min with sustained heating and agitation. After that, the fibre was withdrawn and immediately placed in the GC for desorption and analysis. The GC–MS analysis was carried out using an Agilent 7890 gas chromatography system and an Agilent 5977 mass selective detector. HP‐5 and DB‐WAX (both 30 m × 0.25 mm, i.e., 0.25 mm film thickness) were used to separate the samples. At a flow rate of 1.7 mL/min, helium was employed as the carrier gas, and the GC inlet was set to split‐less mode. The temperature of the injector was 25°C. The temperature program went from 40°C (2 min hold) to 160 at 4°C each minute, then to 28°C at 50°C every minute. Then, using 70‐eV ionisation energy, electron ionisation mode (EI) was adopted. The mass range was 35 to 450 m/z, and the ion source temperature was 23°C. Authentic standards, retention indices (RI) and the NIST 14.0 library were used to determine the volatile chemicals. Compound retention indices (RIs) were calculated by injecting a homologous sequence of straight‐chain alkanes (C6–C30) into a sample.

### 2.6 In Silico Study

#### 2.6.1 Ligand Preparation

To understand the potential health effects of the lactic acid bacteria, we analysed their volatile compounds using a technique called GC–MS. Then, we retrieved the 3D structures of these compounds in a public database called PubChem [26]. Finally, we used software (Avogadro) to refine the 3D structures for further analysis [27].

#### 2.6.2 Protein Preparation

The crystal structure of human DPP4 bound to a new heterocyclic inhibitor was retrieved from the Protein Data Bank (PDB ID: 4A5S). The structure was refined and cleaned using the Discovery Studio [28]. Further optimisation of the cleaned and refined crystal structure was performed through the YASARA dynamics software package [29].

#### 2.6.3 Molecular Docking

The PyRx virtual screening tool was used to carry out molecular docking of each compound found in the bacterium [30]. The compound's energy was minimised and then converted to the PDBQT format, and the grid box center and dimensions (Angstrom) were set to (X:13.5479, Y:26.4378, Z:55.8450) and (X:68.5588 Å, Y:77.0176 Å, Z:69.5275 Å), respectively [31, 32, 33]. Following visualisation of the docked protein–ligand complexes and analysis of the docking poses with BIOVIA Discovery Studio, a nonbonding interaction analysis was carried out using PyMol [34] and BIOVIA Discovery Studio [28]. The best conformations were chosen based on the docking score (in kcal/mol) following the completion of the docking investigation.

#### 2.6.4 Molecular Dynamics Simulation

YASARA dynamics software package in association with the AMBER14 force field was used to simulate the ligand–protein complexes [29]. A series of initial steps have been taken on the docked complexes, including cleaning, optimisation and hydrogen bond networking. In order to create a cubic simulation cell, the TIP3P solvation model was utilised with periodic boundary conditions [35]. A physiological condition of 298 K temperature, 0.9% NaCl concentration and pH 7.4 was established in the simulation cell. Energy minimisation was initially conducted using simulated annealing with the steepest descent method over 5000 cycles. 1.25 fs was set as the simulation time step [36]. The long‐range electrostatic interactions were calculated using the Particle Mesh Ewald (PME) method with the 8 Å cutoff radius [37, 38, 39]. Simulation trajectory data were kept after every 100 ps. The Berendsen thermostat was used to run simulations under constant pressure and temperature for 100 ns. Analysis of the simulation trajectories included calculating the root mean square deviation (RMSD), root mean square fluctuation (RMSF), solvent‐accessible surface area (SASA), radius of gyration and hydrogen bonds [40, 41, 42, 43, 44, 45, 46, 47, 48].

#### 2.6.5 ADMET Analysis

A number of pharmacokinetic software packages were used to predict the pharmacokinetics of the compounds, including SwissADME, admetSAR and pkCSM [49, 50]. This analysis primarily focused on assessing the drug‐likeness of the top‐docked compounds [51].

### 2.7 Data Analysis

After collecting the data, they were reviewed, coded and entered into IBM SPSS statistical software version 22 (SPSS Inc., Chicago, IL). With the help of GraphPad Prism version 8.0, graphs were constructed. Two‐tailed tests were used for statistical analyses, and a *p*‐value of less than 0.05 was considered statistically significant.

## 3 Results

### 3.1 In Vivo Study

#### 3.1.1 Impact of *Lactobacillus plantarum* DMR14 on Blood Sugar Levels in STZ‐Induced Diabetic Mice

The effect of the bacterial strain on STZ‐induced mice was observed over a 21‐day period, with measurements taken every 7 days. Blood glucose levels of STZ‐induced mice treated with *Lactobacillus plantarum* were compared. The results showed a significant reduction in blood sugar levels in the mice treated with *Lactobacillus plantarum* . Notably, on the 21st day, these mice had a 35.03% decrease in blood sugar compared to their initial levels at the beginning of the study.

#### 3.1.2 Impact of *Lactobacillus plantarum* DMR14 on Final Body and Organ Weights

**FIGURE 1:**
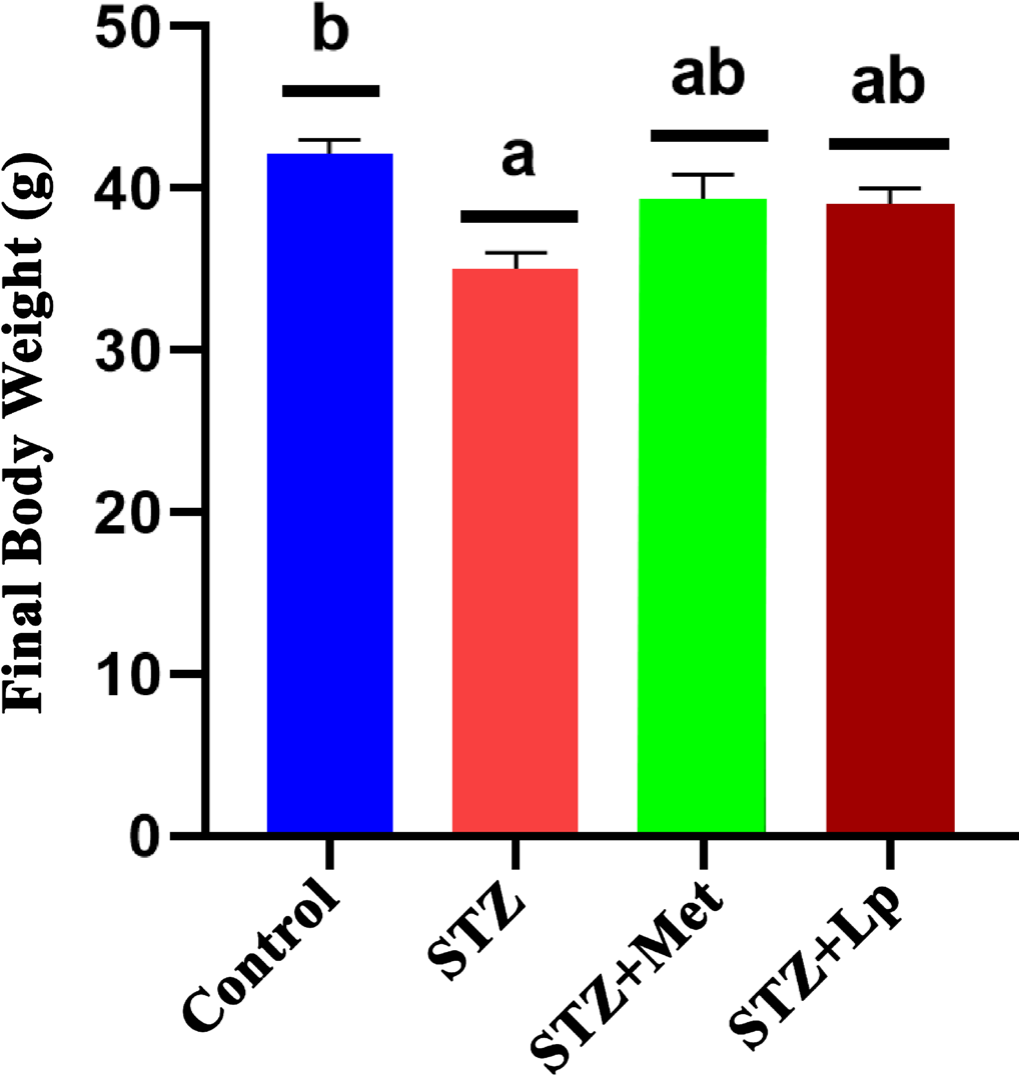
Effects of *Lactobacillus plantarum* DMR14 on final body weight of albino mice. Different letters used to indicate significant differences between the mean ± SD of replications (*n* = 3) at a significance level of *p* < 0.05. (a) *p*‐value was < 0.05 versus normal group; (b) *p* < 0 0.05 versus STZ group. Here, STZ indicates streptozotocin, Met indicates standard drug metformin and Lp indicates the used bacterial strain *Lactobaccilus plantarum* DMR14.

**FIGURE 2:**
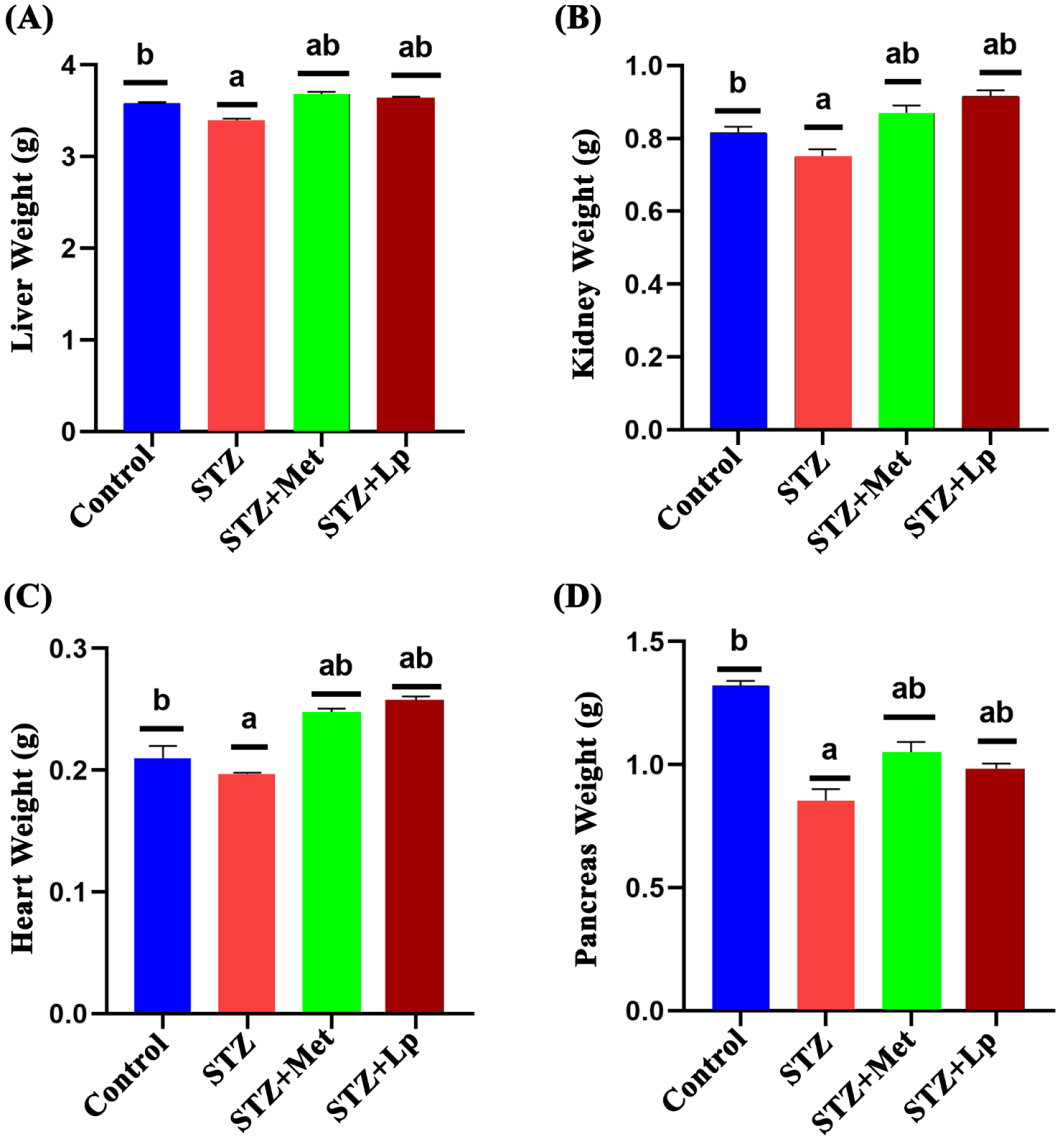
Effects of *Lactobacillus plantarum* DMR14 on organ weight after 21 days of treatment. (A) Liver Weight; (B) Heart Weight; (C) Kidney Weight and (D) Pancreas Weight. Different letters used to indicate significant differences between the mean ± SD of replications (*n* = 3) at a significance level of *p* < 0.05. (a) *p*‐value was < 0.05 versus normal group; (b) *p* < 0.05 versus STZ group. Here, STZ indicates streptozotocin, Met indicates standard drug metformin and Lp indicates the used bacterial strain *Lactobaccilus plantarum* DMR14.

The analysis revealed statistically significant variations (*p* < 0.05) in final body weight and the weight of several organs (liver, kidney, heart and pancreas) between the treatment and control groups. Especially, on the 21st day, all groups experienced an increase body weight, but the STZ‐induced mice had comparatively lower weight gain than the other groups. Figure 1 shows that the mice treated with both metformin and *Lactobacillus plantarum* (Lp) gained weight at a similar rate to the healthy control group. This pattern was also observed for the organs, as shown in Figure 2.

#### 3.1.3 Impact of *Lactobacillus plantarum* Strain DMR14 on Lipid Levels

**FIGURE 3:**
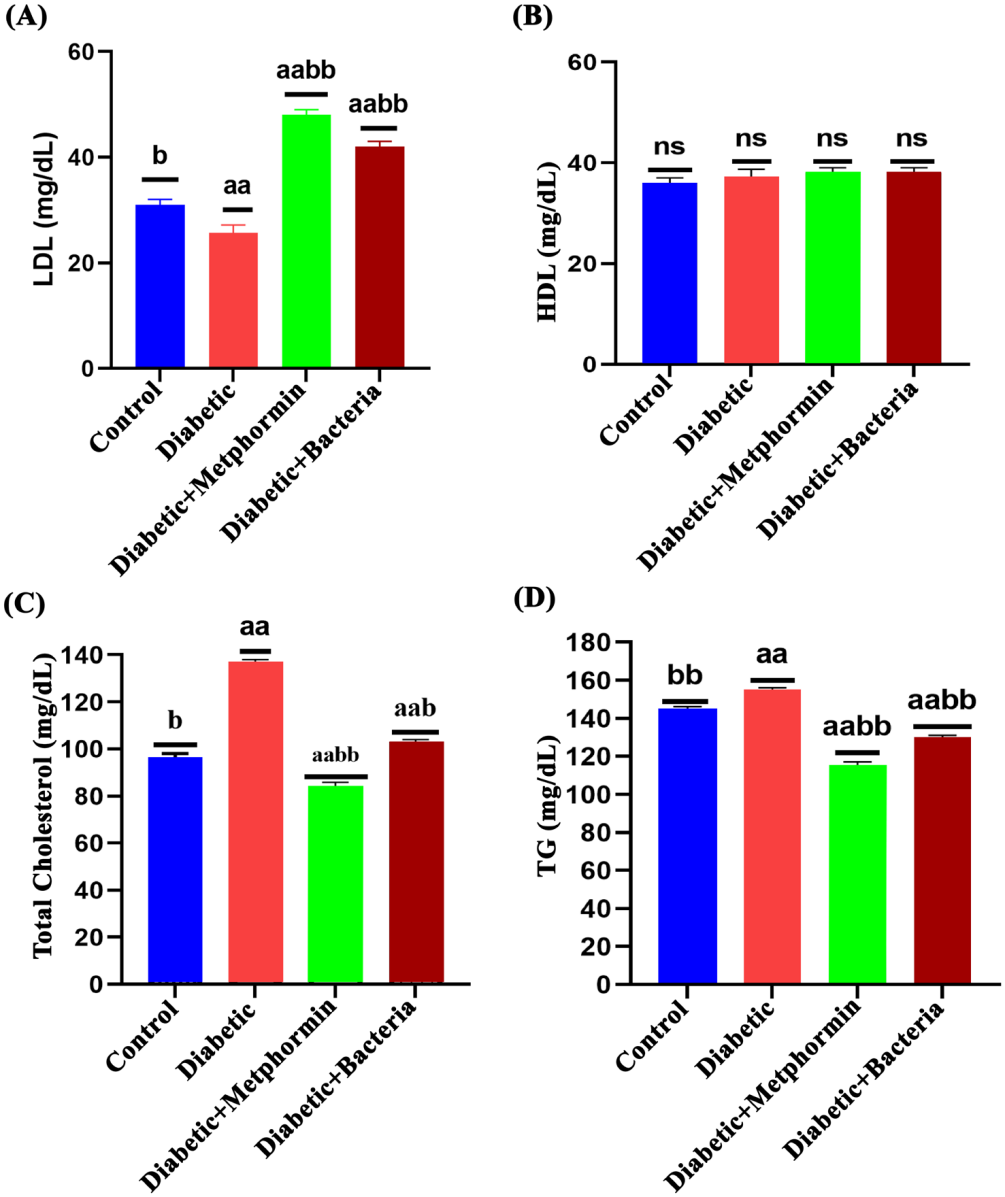
Effect of *Lactobacillus plantarum* DMR14 on the lipid profile of mice (A) Low‐density lipoprotein, (B) High‐density lipoprotein, (C) Total Cholesterol and (D) Triglyceride. Here, STZ indicates streptozotocin, Met indicates standard drug metformin and Lp indicates the used bacterial strain *Lactobaccilus plantarum* DMR14. Different letters used to indicate significant differences between the mean ± SD of replications (*n* = 3) at a significance level of *p* < 0.05.

The treatment resulted in significant reductions in blood levels of triglycerides (TG), total cholesterol (TC) and LDL‐C (low‐density lipoprotein cholesterol), as seen in Figure 3. Interestingly, HDL‐C (high‐density lipoprotein cholesterol) levels remained unchanged.

#### 3.1.4 Impact of *Lactobacillus plantarum* DMR14 on Histopathology

**FIGURE 4:**
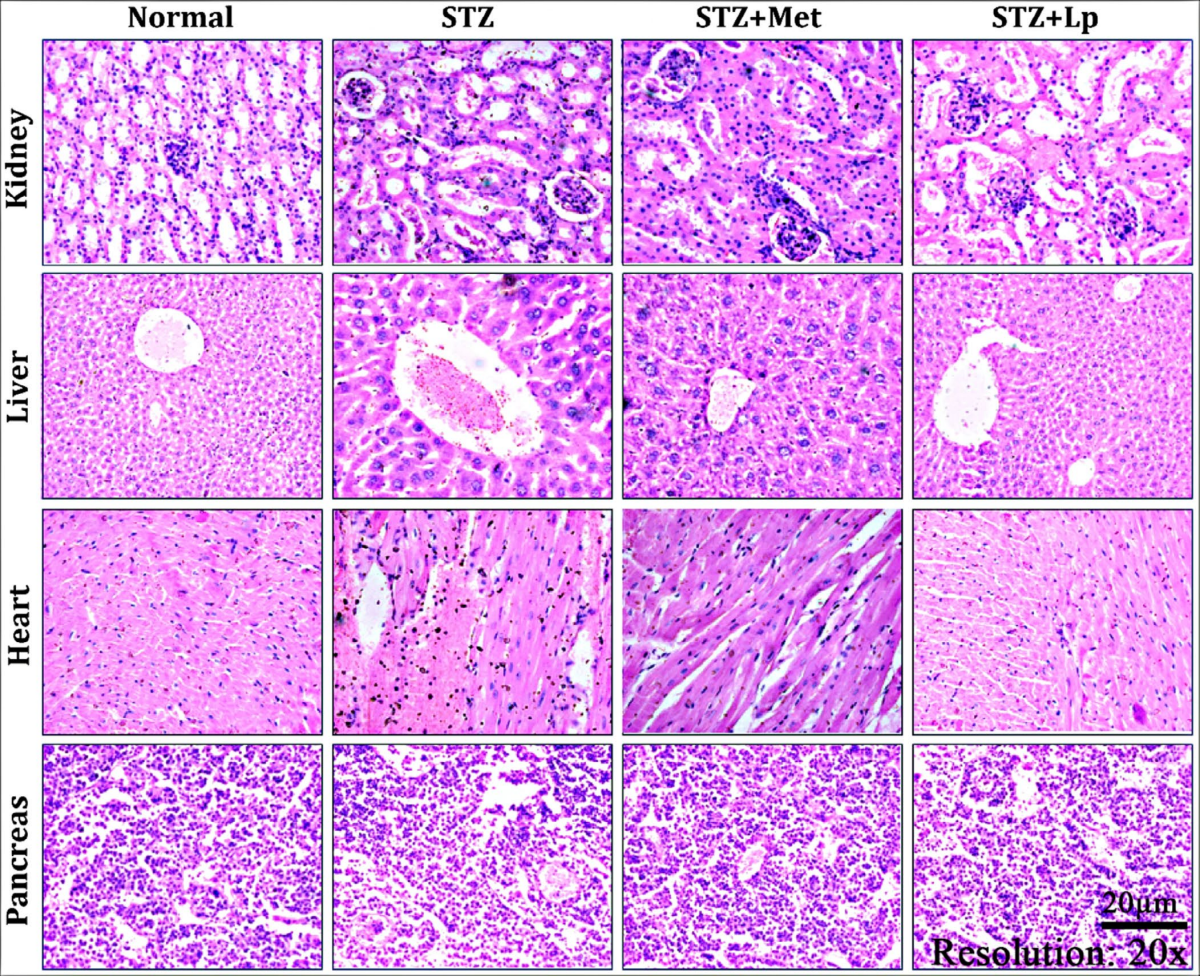
Effect of the *Lactobacillus plantarum* DMR14 treatments on histology of kidney, liver, heart and pancreas of diabetes mice. Here, STZ indicates streptozotocin, Met indicates standard drug metformin and Lp indicates the used bacterial strain *Lactobaccilus plantarum* DMR14.

To assess the effects of the observed biochemical changes on organs, we performed a microscopic examination (histopathological examination) of key tissues like the liver, kidney, heart and pancreas. The healthy mice (nondiabetic) had no signs of damage (histological injuries) in these organs. In contrast, the mice with induced diabetes (STZ‐induced) showed significant damage in various organs. The liver showed swollen veins and cells, the kidney exhibited glomerular shrinkage, the heart had damaged cells, and the pancreas had a loss of cortical tubular and islets of Langerhans, as well as blood in cells (Figure 4). However, treatment with *Lactobacillus plantarum* DMR14 or metformin improved the pathological condition of the organs and resulted in recovery from the histological injuries (Figure 4). The microscopic examination of tissues (histopathological analysis) supports the earlier biochemical test results obtained from both diabetic and healthy mice in this study.

### 3.2 Unveiling the Volatile Fingerprint of *Lactobacillus plantarum* DMR14 Using GC–MS

GC–MS analysis identified a total of 70 volatile compounds produced by the bacteria, which are detailed in Table S1 and Figure S1. Phenol, 3,5‐bis(1,1‐dimethylethyl)—was the most abundant compound, accounting for 11.03% of the total volatiles. Neophytadine was also a significant component, present at 5.17%, followed by another compound identified as Indole at 2.08%.

### 3.3 In Silico Study

Table 3 provides a comprehensive overview of all identified compounds, including their International Union of Pure and Applied Chemistry (IUPAC) names, PubChem IDs (unique identifiers in a public database), molecular weights, retention times during GC–MS analysis (indicating their elution order) and any docking results obtained.

#### 3.3.1 Docking Exploration for Lead Identification

This section describes a computational analysis (molecular docking) performed to investigate how the identified volatile compounds interact with the DPP‐4 receptor‐binding domain (4A5S). This analysis aimed to find potential lead molecules with strong binding affinity (Table S1). The top ten compounds with the most favourable binding energies, ranging from −10.1 to −7.1 kcal/mol, are presented in Table 3. The specific interactions between these compounds and the receptor were identified using Discovery Studio software.

#### 3.3.2 Protein–Ligand Interaction

**FIGURE 5:**
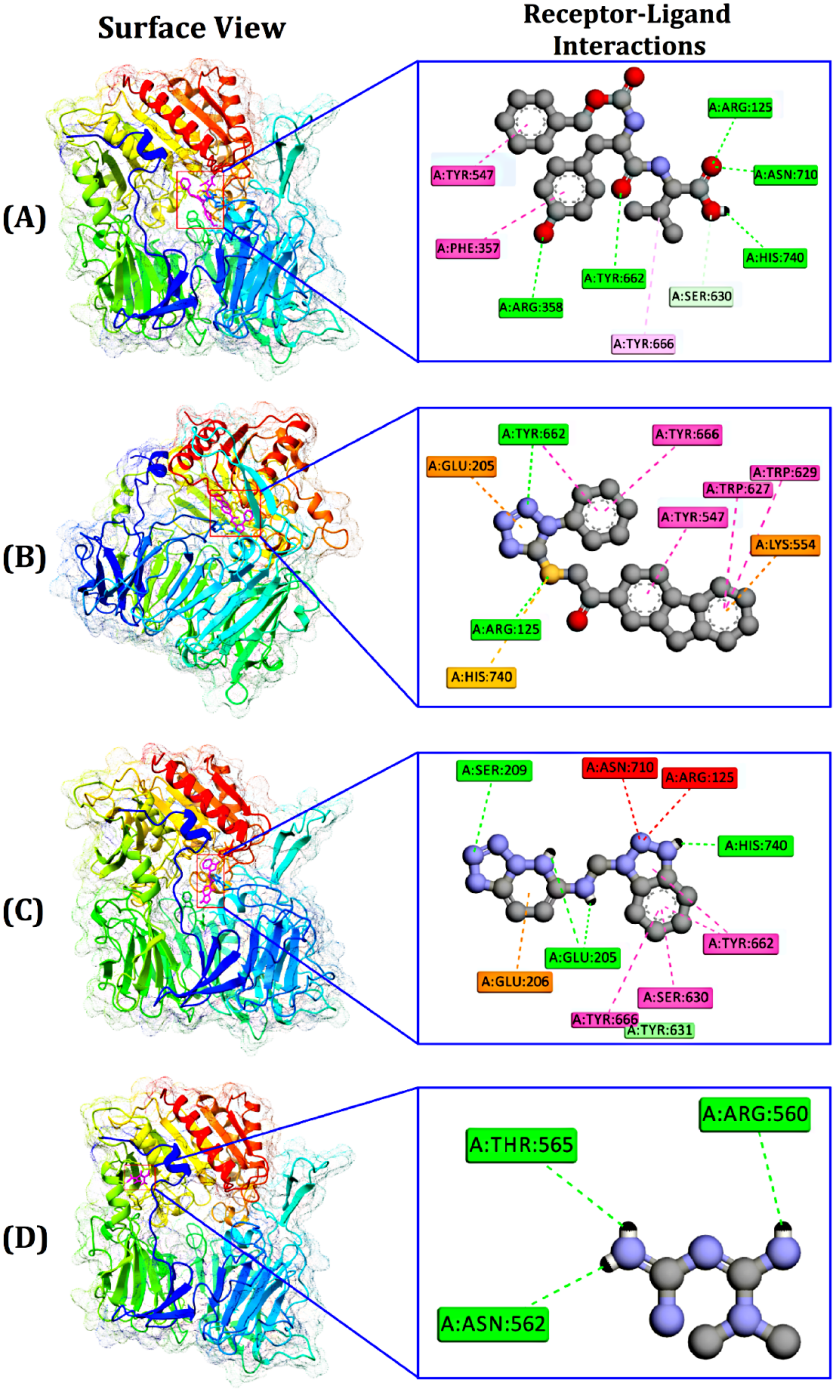
A docking simulation of DPPH (4A5S) protein with N‐carbobenzyloxy‐l‐tyrosyl‐l‐valine (A), −(9H‐Fluoren‐2‐yl)‐2‐(1‐phenyl‐1H‐tetrazol‐5‐ylsulphanyl)‐ethanone ((B), Benzotriazol‐1‐ylmethyl)(tetrazolo[1,5‐b]pyridazin‐6‐yl)amine and 1 (C) and Metformin (D) showing the pose view, and receptor–ligand interactions at 2D view.

The interaction of the small molecules with 4A5S of diabetes had been investigated with bond distance. From Table 4, it is clear that there were two types of bonds, the H‐bond and the hydrophobic bond. For metaphor, there was no hydrophobic bond. The top‐scoring compound, 1‐(9H‐Fluoren‐2‐yl)‐2‐(1‐phenyl‐1H‐tetrazol‐5‐ylsulphanyl)‐ethanone, exhibited two key interactions with the target molecule: two hydrogen bonds formed with specific amino acids in the receptor (Arg125 and Tyr662) as well as four hydrophobic interactions, likely involving nonpolar regions of both the compound and the receptor. Figure 5 displays the surface and 2D views of the binding of the top five small molecules and metformin.

#### 3.3.3 ADMET Analysis

To assess the potential of the top ten shortlisted compounds as drug candidates, their drug‐likeness properties were evaluated using ADMET calculations (Table 5). These calculations consider various factors that influence a drug's behaviour in the body, including absorption, distribution, metabolism, excretion and toxicity (ADMET). One key aspect of drug‐likeness is molecular weight, as stipulated by Lipinski's Rule of Five. This rule suggests that a molecule with a weight below 500 Da (g/mol) is more likely to exhibit good oral bioavailability [52]. The molecular weight of the top ten potential compounds was under 500 g/mol. Additionally, to be a viable drug candidate, a compound should have fewer than ten H‐bond acceptors and no more than five H‐bond donors. All ten compounds adhered to this criterion. The Topological Polar Surface Area (TPSA) for these compounds ranged from Å^2^ to 140 Å^2^, and their gastrointestinal (GI) absorption was high [49]. All of the compounds’ TPSAs were in between the range, and except for one compound, all of them had high GI absorption. While six of the top ten compounds strictly adhered to all criteria of Lipinski's Rule of Five, the remaining four exhibited compliance with most aspects, with only a single violation. It is important to note that exceeding one parameter within Lipinski's Rule does not necessarily disqualify a compound from consideration as a potential drug candidate.

#### 3.3.4 Molecular Dynamics Simulation

**FIGURE 6:**
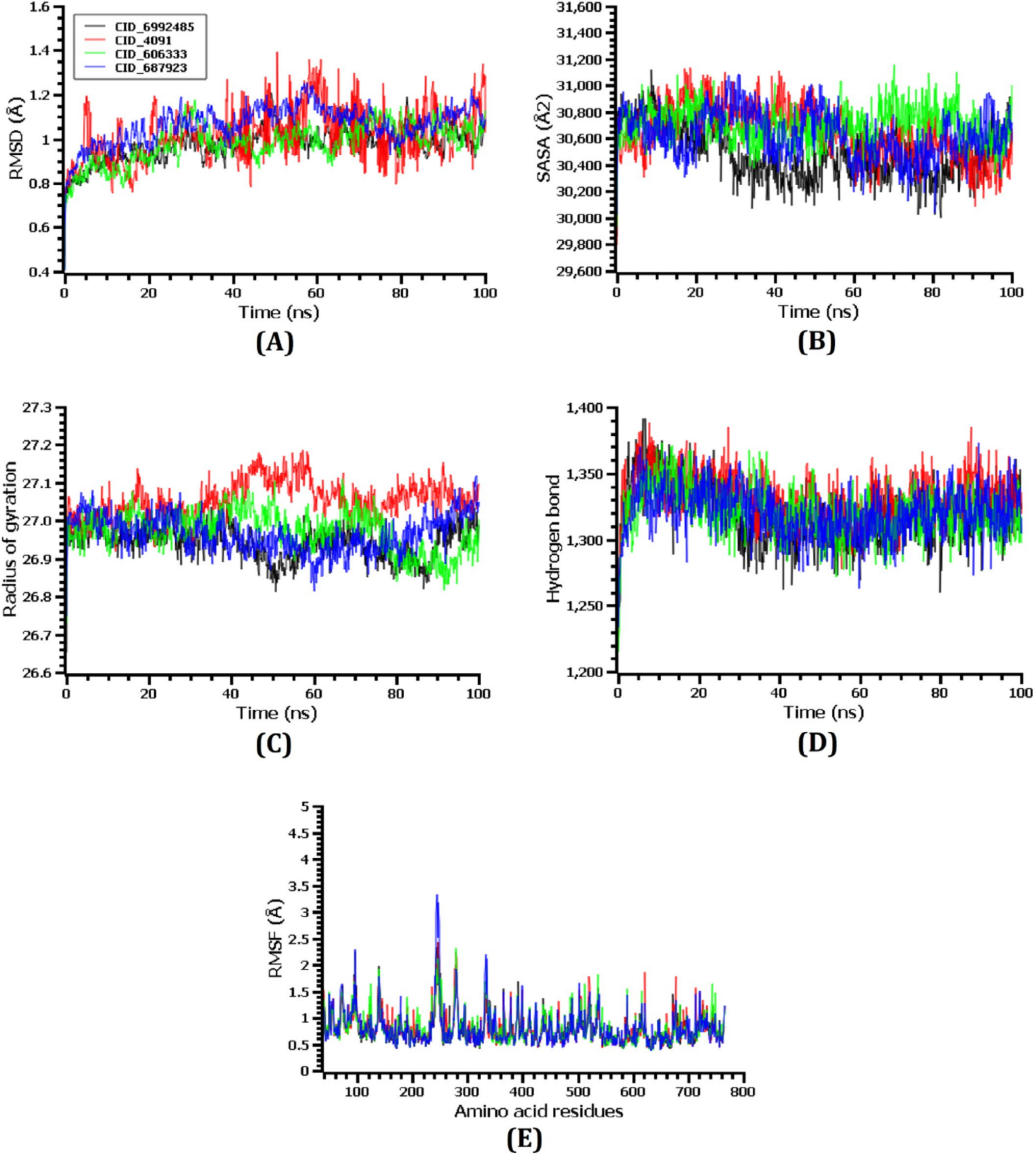
The time‐series analysis of all the simulation trajectories; alphabetically, the RMSD of alpha carbon atoms is presented in (A), SASA is presented in (B), Rg is presented in (C), Hydrogen bonds between complexes is presented in (D), and amino acid residue flexibility is displayed in (E).

The structural stability of the top three ligand–protein complexes, as well as the control drug (Metformin)–protein complex, was evaluated, and the docking potentials for these complexes were examined through molecular dynamics simulations. To assess the stability variations in the protein–ligand complexes, the RMSDs of the C‐alpha atoms were calculated. Figure 6A illustrates the initial increase in RMSD values of CID_6,992,485–DPP4, CID_606,333–DPP4, CID_687,923–DPP4 and CID_4091 (Metformin)–DPP4 complexes due to their instability. A series of upward and inward RMSD trends were observed in the CID_4091 (Metformin)–DPP4 complex during the 100 ns simulation time, indicating its high degree of flexibility. CID_687,923–DPP4 complex exhibited a higher average RMSD rise compared to the other two complexes (except Metformin–DPP4). The RMSD of the CID_687,923–DPP4 complex initially displayed a significant decrease up to 60 ns (ns) of the simulation. This was followed by a stabilisation phase around 75 ns, with minimal fluctuations observed for the remaining 25 ns. The CID_606,333–DPP4 complex exhibited a distinct trend. Its RMSD initially fluctuated inward until 60 ns (ns) before rising steadily around 65 ns. However, it reached a stable state after 70 ns with minimal variations. In contrast, the CID_6,992,485–DPP4 complex achieved stability much faster, stabilising around 50 ns and maintaining minimal fluctuations throughout the simulation. Notably, these stable RMSD profiles observed in all three ligand–protein complexes (excluding Metformin–DPP4) suggest their overall stability during the simulated timeframe [48].

To understand how the interaction with ligand molecules affects the exposed surface area of the DPP4 protein, the researchers compared the Solvent‐Accessible Surface Area (SASA) values for the top three ligand–protein complexes and the control drug–protein complex. A protein's surface area increases when SASA increases, whereas its surface area decreases when SASA decreases [42, 46]. During 55–90 ns, CID_606,333–DPP4 had the highest SASA value compared to the other complexes, suggesting its extended surface area (Figure 6B). In the 20–90 ns simulation period, the CID_6,992,485–DPP4 complex exhibited the lowest SASA value, indicating a decrease in surface area. During the last 40 ns of simulation time, the CID_687,923–DPP4 and CID_4091 (Metformin)–DPP4 complex remained stable with only minor fluctuations after reaching a steady state at around 60 ns.

In addition to SASA, the Radius of Gyration (Rg) values were evaluated of the protein complexes. Rg is a measure of a molecule's compactness or how tightly its atoms are packed together. A higher Rg value generally indicates a more flexible protein complex, while a lower Rg value suggests a more compact and rigid structure [45]. The Rg values of all three ligand–protein complexes (CID_6,992,485–DPP4, CID_606,333–DPP4 and CID_687,923–DPP4) initially exhibited an increase, suggesting a phase of higher flexibility during the early stages of the simulation. However, the initial decrease in Rg value was observed for CID_4091 (Metformin)–DPP4, which represents their initial stiffness. In addition, there were some greater upward and inward Rg trends for the CID_4091 (Metformin)–DPP4 complex during approximately the entire simulation time, indicating its greater flexibility compared to the other three complexes (Figure 6C).

Proteins rely heavily on hydrogen bonds to maintain their structure and stability [40]. Molecular docking simulations revealed the formation of extensive hydrogen bond networks within the complexes of DPP4 bound to the top three ligand molecules (CID_6,992,485, CID_606,333 and CID_687,923) as well as the control drug, Metformin (Figure 6D). This finding suggests that all four compounds established stable interactions with the DPP4 protein, potentially contributing to their inhibitory effects. To further recognise DPP4's versatility across amino acid residues, the RMSFs of the top three ligands along with the control drug and DPP4 protein complexes were scrutinised. An analysis of the Root Mean Square Fluctuation (RMSF) profiles revealed that nearly all amino acid residues within the top three ligand–protein complexes and the control drug–protein complex exhibited values below 3.5 Å (Figure 6E). Lower RMSF values generally correspond to greater complex stability, which aligns with the observation of reduced flexibility in these complexes compared to the free protein [43].

## 4 Discussion

Type 2 diabetes, a chronic condition influenced by both genetics and lifestyle choices like diet, exercise and weight management, might be positively impacted by probiotics. Research suggests that certain probiotic strains, particularly lactic acid bacteria abundant in fermented foods, hold promise for preventing and managing this condition. Notably, specific strains like *Lactobacillus delbrueckii* , *Enterococcus* sp. and *Lactobacillus fermentum* have been linked to reduced blood glucose levels [53, 54, 55]. Although the precise mechanisms by which lactic acid bacteria work are not fully understood, it is believed to be because these bacteria can break down carbohydrates and produce short‐chain fatty acids, which can help improve insulin sensitivity and reduce inflammation in the body.

Previous studies have demonstrated the potential antidiabetic properties of certain bacteria in vivo. To investigate the effectiveness of *Lactobacillus plantarum* DMR14 in managing diabetes, this study employed a well‐established model. In this model, artificial diabetes was induced in mice through a single high dose of streptozotocin (STZ). STZ specifically targets and destroys beta cells in the pancreas, which are responsible for insulin production [56]. Our study demonstrated that oral administration of *Lactobacillus plantarum* DMR14 effectively reduced elevated blood glucose levels in mice with STZ‐induced diabetes, compared to a control group of diabetic mice that did not receive the probiotic (Table 2). The findings from Hasanian, Yang as well as Kim, Seulki also support the antidiabetic activity of *Lactobacillus plantarum* through in vitro and in vivo studies [6, 57, 58].

**TABLE 2:** Effects of *Lactobacillus plantarum* on serum glucose level in STZ‐induced diabetic mice. Data were taken on days 7, 14 and 21. Here, STZ indicates streptozotocin, Met indicates standard drug metformin and Lp indicates the used bacterial strain *Lactobaccilus plantarum*. Data are the mean ± SD for four mice in each group; where significant values are *p* < 0.05.

Groups	Serum glucose level (mmol/L)			
	Before treatment (5 days after injecting STZ)	After treatment		
	On 0 day	On 7th day	On 14th day	On 21st day
Normal	4.7 ± 0.26	4.7 ± 0.25	4.6 ± 0.26	4.6 ± 0.15
STZ	13.4 ± 0.51	14.3 ± 1.52	16.7 ± 2.08	17.3 ± 1.52
STZ + Met	13.9 ± 1.04	9.4 ± 0.92	8.6 ± 0.68	8.1 ± 0.73
STZ + Lp	13.7 ± 0.76	11.00 ± 1.32	9.3 ± 0.15	8.9 ± 0.45

It was interesting to see that the diabetic mice treated with metformin or *Lactobacillus plantarum* DMR14 had a higher final body weight and organ weight compared to untreated diabetic mice (Figures 1, 2). These suggest that these interventions may have a protective effect against the weight loss often seen in individuals with uncontrolled diabetes. This result was also similar to the other research [59]. The rise in total cholesterol and total triglyceride levels in STZ‐induced diabetic mice is also consistent with previous findings, which suggest that liver damage and impaired liver function may contribute to higher blood glucose levels and dyslipidaemia in diabetes [60]. In this study, notable differences in total cholesterol and triglyceride levels were observed among diabetic, nondiabetic and treated diabetic mice (*p* < 0.05; Figure 3). The total cholesterol and triglyceride levels of STZ‐induced diabetic mice were higher than those of nondiabetic and treated diabetic mice. Treatment with *Lactobacillus plantarum* DMR14 significantly reduced total cholesterol by 42.85% and triglycerides by 24.13% in treated diabetic mice compared to untreated diabetic mice (Figure 3). This suggests that probiotics could be an effective complementary therapy for managing dyslipidaemia in diabetes. No significant difference in HDL levels was found, but LDL levels were elevated in STZ‐induced diabetic mice compared to other groups. The elevated LDL levels in the metformin‐ and bacteria‐treated groups, despite unchanged HDL levels, may result from metformin's variable effects on lipid metabolism or the bacteria's influence on bile acid and cholesterol homeostasis. These changes could reflect a transient phase of lipoprotein remodelling or a host‐specific metabolic response. The study's treatment duration might also have captured an intermediate stage, with longer‐term studies needed to clarify these effects. These findings align with previous studies reporting increased total cholesterol, triglycerides and LDL levels in STZ‐induced diabetic mice [60]. Both the induction of diabetes and the treatment interventions may have an impact on lipid metabolism and lipid profiles.

One of the biggest health problems in the world, type‐2 diabetes affects people of all ages and genders. Researchers are now concentrating their efforts on natural sources to find naturally active phytochemicals with fewer side effects and greater antidiabetic efficacy, despite the market's availability of several synthetic oral antidiabetic medications [61]. Despite several in silico studies being conducted to find prospective compounds against diabetes‐associated proteins from plant derivatives, there are no studies that have discovered possible compounds against diabetes from volatile chemicals. There is growing evidence that certain bacterial volatile compounds may have antidiabetic properties. Bacterial volatile compounds are small molecules that bacteria release into the environment, and they play important roles in intercellular communication and modulating host physiology [62]. The identification of volatile compounds produced by probiotic bacteria, such as *Lactobacillus plantarum* DMR14, is an area of growing interest in the scientific community due to their potential health benefits. Dipeptidyl peptidase‐4 (DPP‐4) is one of the known targets for diabetes as it increases the blood glucose level after consuming food [63, 64, 65].

Through molecular docking, the inhibitory action of bacterial substances against dipeptidyl peptidase‐4 (DPP‐4) (PDB: 4A5S) was investigated in the current study. When a compound interacts with a protein's active site, it can be deemed effective against the protein in the context of designing in silico drugs. In this study, we identified three top potential antidiabetic candidates to target protein (4A5S): 1‐(9H‐Fluoren‐2‐yl)‐2‐(1‐phenyl‐1H‐tetrazol‐5‐ylsulphanyl)‐ethanone (with a binding energy of −10.1 kcal/mol), (Benzotriazol‐1‐ylmethyl) (tetrazolo[1,5‐b]pyridazin‐6‐yl)amine (with a binding energy of −9.7 kcal/mol) and N‐carbobenzyloxy‐l‐tyrosyl‐l‐valine (with a binding energy of −8.7 kcal/mol) (Table 3). The majority of the compounds are recommended to be promising drug candidates through assessment of the ADMET and pharmacokinetics properties. Except for one compound that had a high GI absorbance efficacy, all of the top 3 compounds in this study met the requirements for H‐bond donors and recipients (Table 5) and at least four of Lipinski's five guidelines [49]. The suggested compounds could thereby pave the way for the development of completely novel drugs for diabetes.

**TABLE 3:** The compounds name, PubChem ID, molecular weight and binding energy of the top 10 compounds.

Compounds name	PubChem ID	Molecular weight (g/mol)	Binding energy (kcal/mol)
1‐(9H‐Fluoren‐2‐yl)‐2‐(1‐phenyl‐1H‐tetrazol‐5‐ylsulphanyl)‐ethanone	606,333	384.5	−10.1
(Benzotriazol‐1‐ylmethyl) (tetrazolo[1,5‐b]pyridazin‐6‐yl)amine	687,923	267.25	−9.3
N‐carbobenzyloxy‐l‐tyrosyl‐l‐valine	6,992,485	414.5	−8.7
1,8‐Di(4‐nitrophenylmethyl)‐3,6‐diazahomoadamantan‐9‐one	547,088	436.5	−8.6
1‐Ethyl‐2‐[4‐(4‐nitrobenzyl)piperazin‐1‐yl]‐1H‐benzoimidazole	1,462,232	365.4	−8.6
1,1‐Cyclobutanedicarboxamide, 2‐phenyl‐N,N′‐bis(1‐phenylethyl)—	570,689	426.5	−8.0
methanesulphonamide, N‐[2‐[ethyl[4‐[[(2‐hydroxyphenyl)methylene]amino]‐3‐methylphenyl]amino]ethyl]—	3,825,197	375.5	−7.4
7,9‐Di‐tert‐butyl‐1‐oxaspiro(4,5)deca‐6,9‐diene‐2,8‐dione	545,303	276.4	−7.2
2,5‐di‐tert‐Butyl‐1,4‐benzoquinone	17,161	220.31	−7.1
1H‐Fluorene, dodecahydro—	21,972	178.31	−7.1

**TABLE 4:** Type of interactions, interacting residues and bond distance of 4A5S with the top three binding energy compounds and the standard metformin.

SL NO	Compounds name	Hydrogen bond		Hydrophobic bond	
		Residues	Distance (A°)	Residues	Distance (A°)
**1**.	Metformin	Arg 560 Asn562 Thr565	2.17 2.82 2.77		
**2**.	1‐(9H‐Fluoren‐2‐yl)‐2‐(1‐phenyl‐1H‐tetrazol‐5‐ylsulphanyl)‐ethanone	Arg125 Tyr662	2.56 2.47	Trp 629 Trp 627 Tyr547 Tyr666	5.82 5.26 4.83 4.94
**3**.	(Benzotriazol‐1‐ylmethyl) (tetrazolo[1,5‐b]pyridazin‐6‐yl)amine	Phe695 Met689	3.90 5.79	Leu723 Val723 Val726	4.67 4.27 4.71
**4**.	N‐carbobenzyloxy‐l‐tyrosyl‐l‐valine	His740 Arg125 Asn710 Tyr662 Arg358	4.71 6.73 4.66 7.80 6.94	Tyr666 Phe357 Tyr547	5.82 5.04 4.78

**TABLE 5:** The ADMET calculation of the top ten compounds. Here, TPSA—topological surface area; BBB—blood–brain barrier.

Physical properties						Lipophilicity	Water solubility	Pharmacokinetics	Drug likeliness			
Compounds	Molecular weight (g/mol)	Num. H‐bond acceptors	Num. H‐bond donors	Fraction Csp3	TPSA (Å^2^)	Log P_o/w_ (XLOGP3)	Log S (ESOL)	GI absorption	BBB	Lipinski/Ghose/veber/Egan/Muegge	Bioavailability	Recommended
1‐(9H‐Fluoren‐2‐yl)‐2‐(1‐phenyl‐1H‐tetrazol‐5‐ylsulphanyl)‐ethanone	384.5	6	0	0.09	85.97	5.10	−5.71	High	No	L, G, V, E	0.55	Yes
(Benzotriazol‐1‐ylmethyl) (tetrazolo[1,5‐b]pyridazin‐6‐yl)amine	267.25	6	1	0.09	98.71	1.11	−2.66	High	No—	L, G, V, E, M	0.55	Yes
N‐carbobenzyloxy‐l‐tyrosyl‐l‐valine	414.5	6	4	0.32	124.96	2.52	−3.50	High	No	L,G,E,M	0.56	Yes
1,8‐Di(4‐nitrophenylmethyl)‐3,6‐diazahomoadamantan‐9‐one	436.5	7	0	0.43	115.19	2.59	−4.06	High	No	L, G, V, E, M	0.55	Yes
1‐Ethyl‐2‐[4‐(4‐nitrobenzyl)piperazin‐1‐yl]‐1H‐benzoimidazole	365.4	4	0	0.35	70.12	3.33	−4.28	High	Yes	L, G, V, E, M	0.55	Yes
1,1‐Cyclobutanedicarboxamide, 2‐phenyl‐N,N′‐bis(1‐phenylethyl)—	426.5	2	2	0.29	58.20	5.06	−5.49	High	Yes	L, G, V, E	0.55	Yes
methanesulphonamide, N‐[2‐[ethyl[4‐[[(2‐hydroxyphenyl)methylene]amino]‐3‐methylphenyl]amino]ethyl]—	375.5	5	2	0.32	90.38	2.82	−3.76	High	No	L, G, V, E, M	0.55	Yes
7,9‐Di‐tert‐butyl‐1‐oxaspiro(4,5)deca‐6,9‐diene‐2,8‐dione	276.4	3	0	0.65	43.37	3.81	−3.82	High	Yes	L, G, V, E, M	0.55	Yes
2,5‐di‐tert‐Butyl‐1,4‐benzoquinone	220.3	2	0	0.57	34.14	4.42	−3.86	High	Yes	L, G, V, E, M	0.55	Yes
1H‐Fluorene, dodecahydro—	178.31	0	0	1.00	0.00	5.45	−4.38	Low	Yes	L, G, V, E	0.55	Yes

## 5 Conclusions

In conclusion, this research investigated the antidiabetic potential of *Lactobacillus plantarum* DMR14 using both in vivo and in silico methods. The in vivo results demonstrated that treatment with *Lactobacillus plantarum* DMR14 in diabetic mice led to improvements in organ health, a reduction in blood glucose levels and enhancement of metabolic indicators. Furthermore, in silico docking studies identified several bioactive compounds, including 1‐(9H‐Fluoren‐2‐yl)‐2‐(1‐phenyl‐1H‐tetrazol‐5‐ylsulphanyl)‐ethanone, (Benzotriazol‐1‐ylmethyl) (tetrazolo[1,5‐b]pyridazin‐6‐yl)amine and N‐carbobenzyloxy‐l‐tyrosyl‐l‐valine, which may contribute to the observed enzyme inhibitory activity. These findings highlight the presence of promising antidiabetic compounds in *Lactobacillus plantarum* DMR14, warranting further investigation into their efficacy as potential therapeutic agents for managing diabetes mellitus.

## Author Contributions

**Shirmin Islam:** conceptualization (equal), data curation (equal), formal analysis (equal), methodology (equal), writing – original draft (equal). **Suvro Biswas:** data curation (equal), formal analysis (equal), methodology (equal), writing – original draft (equal). **Md. Ariful Islam:** data curation (equal), formal analysis (equal), investigation (equal), methodology (equal). **Jui Biswas:** data curation (equal), formal analysis (equal), investigation (equal). **Amit Kumar Dutta:** formal analysis (equal), methodology (equal), validation (equal). **Golam Gaus Mohiuddin:** data curation (equal), formal analysis (equal), methodology (equal). **Md. Abu Saleh:** conceptualization (equal), funding acquisition (equal), project administration (equal), supervision (equal), writing – review and editing (equal). **Shahriar Zaman:** project administration (equal), resources (equal), writing – review and editing (equal).

## Ethics Statement

In this investigation, all investigational protocols, including animal experiments, were permitted by the Institute of Biological Sciences, University of Rajshahi's Institutional Animal, Medical Ethics, Biosafety and Biosecurity Committee (IAMEBBC) for Experiments on Animals, Humans, Microorganisms and Living Natural Sources (Memo no: 249 (35)/320/IAMEBBC/IBSc; Dated: November 16,2022), according to the Basel Declaration.

## Conflicts of Interest

The authors declare no conflicts of interest.

## Supporting information


Data S1.

## Data Availability

The datasets used and/or analysed during the current study are available from the corresponding author upon reasonable request.
