# Supplementary material for: Multifaceted Analysis of Lactobacillus plantarum DMR14 Reveals Promising Antidiabetic Properties Through In Vivo Assays and Molecular Simulations
Source: J Cell Mol Med. 2025 Jan 26;29(2):e70347. doi: 10.1111/jcmm.70347 (PMC11769970; doi:10.1111/jcmm.70347)
Supplement: Supplementary file 1 — Data S1. [file JCMM-29-e70347-s001.docx]

**Multifaceted Analysis of *Lactobacillus plantarum* DMR14 Reveals Promising Antidiabetic Properties Through *In Vivo* Assays and Molecular Simulations**

**Shirmin Islam^a^ ǀ Suvro Biswas^a^ ǀ Md. Ariful Islam^a^ ǀ Jui Biswas^a^ ǀ Amit Kumar Dutta^b^ ǀ Golam Gaus Mohiuddin^c^ ǀ Md. Abu Saleh^a,*^ ǀ Shahriar Zaman^a^**

**^a^Microbiology Laboratory, Department of Genetic Engineering and Biotechnology, University of Rajshahi, Rajshahi-6205, Bangladesh**

**^b^Department of Microbiology, University of Rajshahi, Rajshahi-6205, Bangladesh**

**^c^Department of Pharmacy, Noakhali Science & Technology University, Sonapur, Noakhali-3814**

***Corresponding author: Md. Abu Saleh, Professor, Department of Genetic Engineering and Biotechnology, University of Rajshahi, Bangladesh**

**Email:** [**saleh@ru.ac.bd**](mailto:saleh@ru.ac.bd)

**Phone: +8801716731747**

**Fax: 02588866364**

**Table S1: Name, Pubchem ID, molecular weight, retention time, area percentage, and docking score of the identified compounds.**

|  | **Compound Names** | **PubChem ID** | **Molecular Weight (g/mol)** | **R.T.** | **AREA%** | **4A5S** |
| --- | --- | --- | --- | --- | --- | --- |
|  | **Metphormin** | **4091** | **129.16** |  |  | -5.2 |
| **L-1** | **1,8-Di(4-nitrophenylmethyl)-3,6-diazahomoadamantan-9-one** | **547088** | **436.5** | **1.025** | **0.1** | **-8.6** |
| **L-2** | 1-Butanol, 3-methyl- | 31260 | 88.15 | 1.215 | 0.03 | -4.4 |
| **L-3** | Propane, 1-chloro-2,2-dimethyl- | 12956 | 106.5 | 5.323 | 0.43 | -3.9 |
| **L-4** | n-Amyl isovalerate | 95978 | 172.26 | 5.44 | 0.09 | -5.2 |
| **L-5** | 1-[1-(2,2-Dichlorovinylimino)-2,2-dimethylpropyl]-3-(p-tolyl)thiourea | 2252469 | 344.3 | 5.58 | 0.03 | -6.9 |
| **L-6** | Acetic acid, [4-(1,1-dimethylethyl)phenoxy]-, methyl ester | 101331 | 222.28 | 5.8 | 0.11 | -6.2 |
| **L-7** | 2-Heptanone | 8051 | 114.19 | 7.581 | 0.5 | -4.4 |
| **L-8** | 2-Nonanone | 13187 | 142.24 | 7.74 | 0.12 | -4.6 |
| **L-9** | 2-Butyl-3-methylpyrazine | 85228 | 150.22 | 8.21 | 0.1 | -5.2 |
| **L-10** | **1-(9H-Fluoren-2-yl)-2-(1-phenyl-1H-tetrazol-5-ylsulfanyl)-ethanone** | **606333** | **384.5** | **8.37** | **0.05** | **-10.1** |
| **L-11** | **1,1-Cyclobutanedicarboxamide, 2-phenyl-N,N'-bis(1-phenylethyl)-** | **570689** | **426.5** | **10.192** | **0.12** | **-8.0** |
| **L-12** | trans-3-Caren-2-ol | 576906 | 152.23 | 10.255 | 0.07 | -6.0 |
| **L-13** | 1,8-Nonadien-3-ol | 545753 | 140.22 | 10.344 | 0.66 |  |
| **L-14** | Eucalyptol | 2758 | 154.25 | 10.42 | 0.16 | -6.0 |
| **L-15** | **N-carbobenzyloxy-l-tyrosyl-l-valine** | **6992485** | **414.5** | **10.475** | **0.09** | **-8.7** |
| **L-16** | 2-Methyl-3-isopropylpyrazine | 519203 | 136.19 | 10.633 | 0.48 | -5.5 |
| **L-17** | cis-Chrysanthenyl propionate | 529752 | 208.30 | 10.846 | 0.15 | -6.1 |
| **L-18** | 1-Octanol | 957 | 130.23 | 10.192 | 0.12 | -4.5 |
| **L-19** | Pyrazine, 3-ethyl-2,5-dimethyl- | 25916 | 136.19 | 11.237 | 0.37 | -5.5 |
| **L-20** | **methanesulfonamide, N-[2-[ethyl[4-[[(2hydroxyphenyl)methylene]amino]-3-methylphenyl]amino]ethyl]-** | **3825197** | **375.5** | **11.385** | **0.33** | **-7.4** |
| **L-21** | n-Dodecyl thioglycolate | 19546 | 260.44 | 11.6 | 0.39 | -5.3 |
| **L-22** | 5-Ethyl-3-nonanol | 519707 | 172.31 | 11.845 | 0.03 |  |
| **L-23** | trans-3-Caren-2-ol | 576906 | 152.23 | 11.929 | 0.25 | -6.0 |
| **L-24** | 1,3-Cyclopentadiene, 1,2,3,4-tetramethyl-5-methylene- | 144751 | 134.22 | 12.003 | 0.59 | -5.5 |
| **L-25** | 1,3,3-Trimethyl-2-(2-methyl-cyclopropyl)-cyclohexene | 595941 | 178.31 | 12.269 | 0.05 | -6.8 |
| **L-26** | Cyclopropanemethanol, 2-methyl-2-(4-methyl-3-pentenyl)- | 549579 | 168.28 | 12.376 | 0.41 | -5.5 |
| **L-27** | 1,3-Cyclohexadiene, 1,2,3,4,5,6-hexamethyl- | 591022 | 164.29 | 12.56 | 0.51 | -6.6 |
| **L-28** | Octane, 3-ethyl-2,7-dimethyl- | 537329 | 170.33 | 12.68 | 0.06 | -5.5 |
| **L-29** | **(Benzotriazol-1-ylmethyl)(tetrazolo[1,5-b]pyridazin-6-yl)amine** | **687923** | **267.25** | **12.74** | **0.03** | **-9.3** |
| **L-30** | 1-Nonanol | 8914 | 144.25 | 12.805 | 0.32 | -4.9 |
| **L-31** | Cyclohexene, 3,5,5-trimethyl- | 79132 | 124.22 | 12.855 | 0.28 | -5.9 |
| **L-32** | Neophytadiene | 10446 | 278.5 | 12.933 | 5.17 | -5.9 |
| **L-33** | 2-Piperidinone | 12665 | 99.13 | 12.999 | 1.28 | -4.8 |
| **L-34** | Tetradecane, 1-chloro- | 17043 | 232.83 | 13.271 | 0.52 | -4.9 |
| **L-35** | 2-Tridecenal, (E)- | 5283363 | 196.33 | 13.365 | 0.04 | -5.3 |
| **L-36** | Benzothiazole | 7222 | 135.19 | 13.848 | 1.11 | -5.6 |
| **L-37** | 2-Azido-2,4,4,6,6-pentamethylheptane | 546203 | 546203 | 14.001 | 0.09 | -6.0 |
| **L-38** | Cyclohexane, 1,2,3,4,5,6-hexaethyl- | 519497 | 252.5 | 14.06 | 0.24 | -4.9 |
| **L-39** | Benzene, (3-methyl-2-butenyl)- | 20572 | 146.23 | 14.145 | 0.02 | -6.4 |
| **L-40** | 1-Decanol | 8174 | 158.28 | 14.401 | 0.17 | -5.2 |
| **L-41** | 4-Octanone | 11516 | 128.21 | 14.435 | 0.08 | -4.6 |
| **L-42** | 2-(2-Methylpropyl)-3-(1-methylethyl)pyrazine | 585737 | 178.27 | 14.524 | 0.15 | -5.9 |
| **L-43** | Benzene, pentamethyl- | 12784 | 148.24 | 14.61 | 0.05 | -6.5 |
| **L-44** | Indole | 798 | 117.15 | 14.842 | 2.08 | -6.1 |
| **L-45** | Nonane, 5-(2-methylpropyl)- | 545936 | 184.36 | 15.145 | 0.02 | -5.7 |
| **L-46** | Pentadecanal- | 17697 | 226.4 | 15.555 | 0.08 | -5.0 |
| **L-47** | 3-(2-Hydroxy-cyclopentylidene)-2-methyl-propionic acid | 5369050 | 170.21 | 15.813 | 0.14 | -6.1 |
| **L-48** | **1-Ethyl-2-[4-(4-nitrobenzyl)piperazin-1-yl]-1H-benzoimidazole** | **1462232** | **365.4** | **16.015** | **0.1** | **-8.6** |
| **L-49** | 2,4,4,6,6,8,8-Heptamethyl-1-nonene | 545785 | 224.42 | 16.15 | 0.07 | -5.5 |
| **L-50** | Hexadecane | 11006 | 226.44 | 16.278 | 0.14 | -5.3 |
| **L-51** | Dodecanal | 8194 | 184.32 | 16.43 | 0.06 | -4.8 |
| **L-52** | 2-Cyclopenten-1-one, 2-pentyl- | 117549 | 152.23 | 16.484 | 0.07 | -5.7 |
| **L-53** | Z,Z-2,5-Pentadecadien-1-ol | 5364952 | 224.38 | 16.563 | 0.12 | -5.6 |
| **L-54** | Cyclohexanemethanol, 5-t-butyl-2-hydroxy- | 546158 | 186.29 | 16.652 | 0.06 | -6.4 |
| **L-55** | trans-3(10)-Caren-2-ol | 572861 | 152.23 | 16.831 | 0.12 | -5.9 |
| **L-56** | Cyclopentanone, 3-(3-hydroxy-1-propenyl)- | 5367757 | 140.18 | 16.895 | 0.13 | -5.3 |
| **L-57** | 3-t-Butyl-oct-6-en-1-ol | 5365030 | 184.32 | 17.016 | 0.26 | -5.7 |
| **L-58** | **2,5-di-tert-Butyl-1,4-benzoquinone** | **17161** | **220.31** | **17.23** | **0.21** | **-7.1** |
| **L-59** | 1s,4R,7R,11R-1,3,4,7-Tetramethyltricyclo[5.3.1.0(4,11)]undec-2-en-8-one | 595401 | 218.33 | 17.348 | 0.09 | -6.7 |
| **L-60** | Hexanoic acid, 3,5,5-trimethyl-, oct-3-en-2-yl ester | 91693534 | 268.4 | 17.4 | 0.05 | -5.4 |
| **L-61** | Z,Z-2,5-Pentadecadien-1-ol | 5364952 | 224.38 | 17.512 | 0.44 | -5.6 |
| **L-62** | **1H-Fluorene, dodecahydro-** | **21972** | **178.31** | **17.718** | **1.07** | **-7.1** |
| **L-63** | Phenol, 3,5-bis(1,1-dimethylethyl)- | 70825 | 206.32 | 17.767 | 11.03 | -6.9 |
| **L-64** | 2-Isopropyl-5-methyl-1-heptanol | 545941 | 172.31 | 18.253 | 0.03 | -5.6 |
| **L-65** | Sulfurous acid, hexyl octyl ester | 6420812 | 278.45 | 18.115 | 0.04 | -5.2 |
| **L-66** | 2-Diethylamino-4-phenylthiooct-2-ene nitrile | 5375312 | 302.5 | 20.124 | 0.02 | -5.6 |
| **L-67** | trans-2-undecenoic acid | 5282728 | 184.27 | 20.32 | 0.05 | -5.5 |
| **L-68** | 5,8,11,14-Eicosatetraynoic acid | 1780 | 296.4 | 20.375 | 0.02 | -6.2 |
| **L-69** | 1,2-Benzenedicarboxylic acid, bis(2-methylpropyl) ester | 6782 | 278.34 | 23.405 | 0.03 | -6.2 |
| **L-70** | **7,9-Di-tert-butyl-1-oxaspiro(4,5)deca-6,9-diene-2,8-dione** | **545303** | **276.4** | **24.282** | **0.84** | **-7.2** |

**
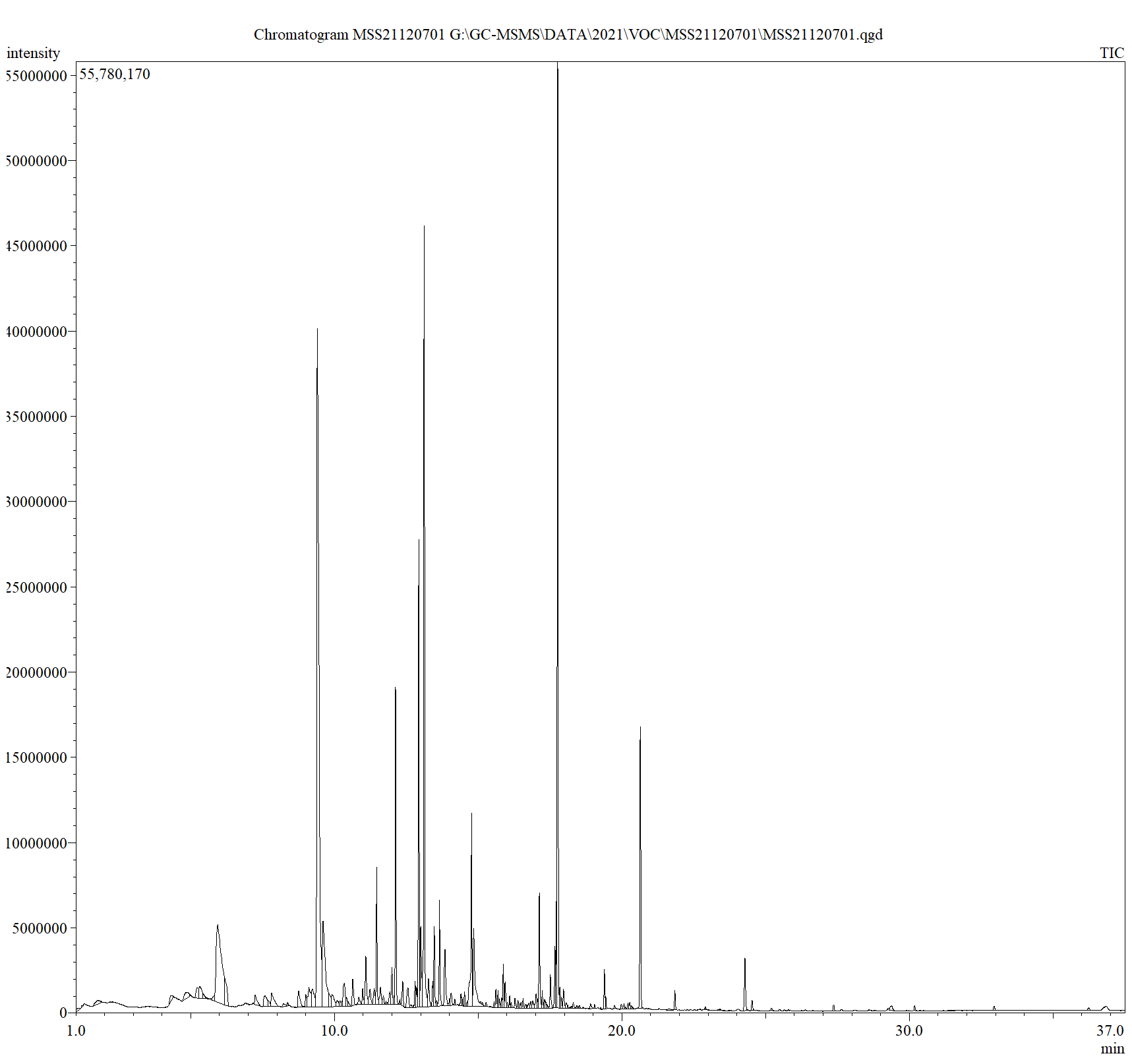
Figure S1: GC-MS chromatogram of the bacterial volatile compounds.**
